# Multigenerational Exposure to Uranium Changes Sperm Metabolome in Rats

**DOI:** 10.3390/ijms23158349

**Published:** 2022-07-28

**Authors:** Stéphane Grison, Audrey Legendre, Ljubica Svilar, Christelle Elie, Dimitri Kereselidze, Céline Gloaguen, Philippe Lestaevel, Jean-Charles Martin, Maâmar Souidi

**Affiliations:** 1 Institut de Radioprotection et de Sûreté Nucléaire, PSE-SANTE, 92260 Fontenay-aux-Roses, France; audrey.legendre@irsn.fr (A.L.); christelle.elie@irsn.fr (C.E.); dimitri.kereselidze@universite-paris-saclay.fr (D.K.); celine.gloaguen@irsn.fr (C.G.); philippe.lestaevel@irsn.fr (P.L.); maamar.souidi@irsn.fr (M.S.); 2 C2VN, CRIBIOM, Aix Marseille Université, 13007 Marseille, France; ljubica.svilar@univ-amu.fr; 3 C2VN, INRAE, INSERM, BIOMET, Aix Marseille Université, 13007 Marseille, France; jean-charles.martin@univ-amu.fr

**Keywords:** uranium, multigenerational, low-dose, chronic exposure, sperm, metabolomic

## Abstract

Male infertility is a major public health issue that can be induced by a host of lifestyle risk factors such as environment, nutrition, smoking, stress, and endocrine disruptors. Regarding the human population exposed to uranium, it is necessary to explore these effects on male reproduction in multigenerational studies. The sensitivity of mass spectrometry (MS)-based methods has already proved to be extremely useful in metabolite identification in rats exposed to low doses of uranium, but also in human sperm. We applied this method to rat sperm over three generations (F0, F1 and F2) with multigenerational uranium exposure. Our results show a significant content of uranium in generation F0, and a reduction in the pregnancy rate only in generation F1. Based on principal component analysis (PCA), we observed discriminant profiles between generations. The partial least squares discriminant analysis (PLS-DA) of the 48 annotated variables confirmed that parental exposure of generation F0 (during both the preconceptional and prenatal periods) can have metabolic effects on spermatozoa for the next two generations. Metabolomics applied to epididymal spermatozoa is a novel approach to detecting the multigenerational effects of uranium in an experimental model, but could be also recommended to identify potential biomarkers evaluating the impact of uranium on sperm in exposed infertile men.

## 1. Introduction

Infertility is a major public health issue, affecting 8–12% of couples worldwide and associated with males in more than half of all cases [1]. In addition, in 30–40% of infertility cases, semen analyses and physical examinations fail to identify the etiology of the dysfunction. These infertility cases are then classified as idiopathic [2]. However, the impact of entropic pollution on ecosystems and human health, especially fertility, is a major concern for scientists and the public, as different pollutants have already been associated with secondary reproductive effects, e.g., endocrine disruptors [3], air pollution [4,5,6] and noise [7]. Thus, the effects of environmental exposure to radionuclides such as uranium on reproduction have become a growing societal concern [8,9,10].

Our literature review identified many experimental studies focusing on different species and demonstrating a range of biological effects for uranium exposure on male or female reproductive functions and fertility [11,12,13,14,15,16,17]. The International Commission on Radiological Protection (ICRP) recently highlighted the need to develop studies to better understand the effects of ionizing radiation over several generations [18]. In addition, the World Health Organization (WHO) supports all studies which could increase knowledge of the effects of environmental pollutants and radiation on the different developmental stages of human life, as well as the multigenerational and intergenerational effects of exposure to such pollutants [19,20].

WHO promotes the development of new tools to increase the sensitivity of diagnoses and improve fertility treatments [21,22]. Proteomics and metabolomics can be used in the field of andrology to help overcome the limitations of standard semen analyses (pH, volume, concentration, motility, morphology, etc.) [23,24,25,26]. In fact, metabolomics are also considered to be more closely related to the actual phenotype than either transcriptomics or proteomics, as they can be used to directly monitor biochemical activity [27,28]. Metabolomics have already demonstrated their ability to identify the mechanisms involved in male infertility by monitoring the metabolomes of human and rodent sperm [29,30]. Mass spectrometry-based methods have already proved to be extremely useful in identifying metabolites in human sperm in case of fertility problems or in asthenozoospermic patients [24,31]. To the best of our knowledge, there are no published reports on the application of MS-based methods to rat sperm with the exception of a few proteome-based studies [32,33].

To better understand the effects of uranium exposure, metabolomics have already been used with various biofluids (blood, urine, cerebrospinal fluid and have demonstrated their ability to detect the effects of low concentrations on rat species [34,35]. Some metabolomic signatures associated with uranium exposure have been demonstrated and used to establish some metabolite fingerprints in human and animals, such as tryptophan and nicotinamide pathways [34,36,37]. The objective of the present experiment is to use a previously published multigenerational study to highlight various biological effects and the metabolomic effects of uranium exposure on rat sperm. Spermatozoa are produced during spermatogenesis in an extremely differentiated cell with very marked genetic, cellular, functional and chromatin changes compared to other cells. This cell delivers the paternal genome to the oocyte and plays profound roles in fertility, embryo development and heredity [38]. The spermatozoa mature while transiting through the epididymis as they acquire different membrane and cellular functionalities [39]. In our study, we focused on rat spermatozoa derived from the epididymis.

First, we aimed to detect the metabolomic signature associated with uranium exposure in rat epididymal sperm for each generation. Then, we identified the metabolites in each generation and, finally, proposed some new markers with biological functions which could affect the metabolic quality of rat sperm.

## 2. Results

### 2.1. Effects on Fertility

**Figure 1:**
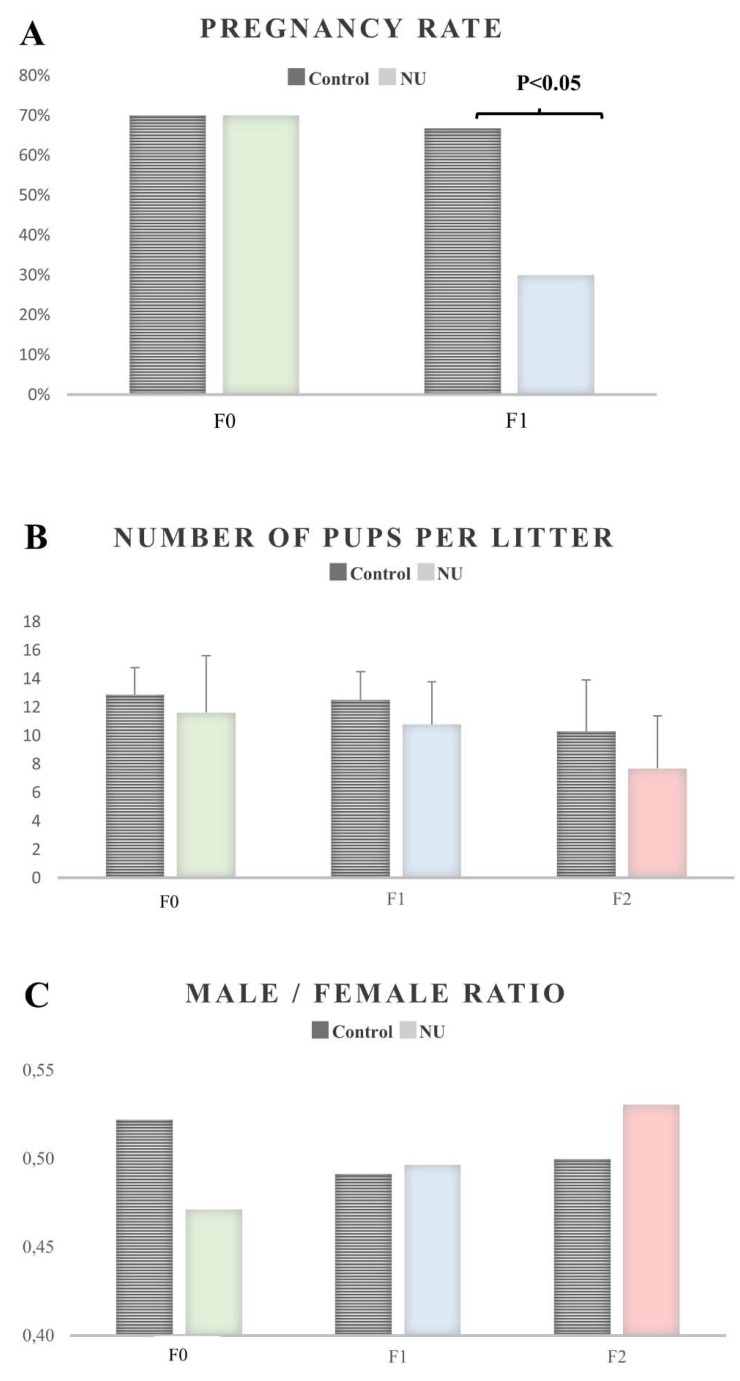
Effects of exposure to uranium on fertility: (**A**) pregnancy rate, (**B**) number of pups per litter and (**C**) sex ratio, were evaluated. Significant effects are defined as *p* < 0.05; n = 17–20 per group.

The pregnancy rate in generation F1 was reduced to 30% in the natural uranium (NU) exposed group in comparison with 70% success in the control group (*p* < 0.05) (Figure 1A). No difference was observed between the successive generations in terms of the number of pups per litter and male/female ratio (Figure 1B,C).

### 2.2. Uranium Quantification in Epididymis

**Figure 2:**
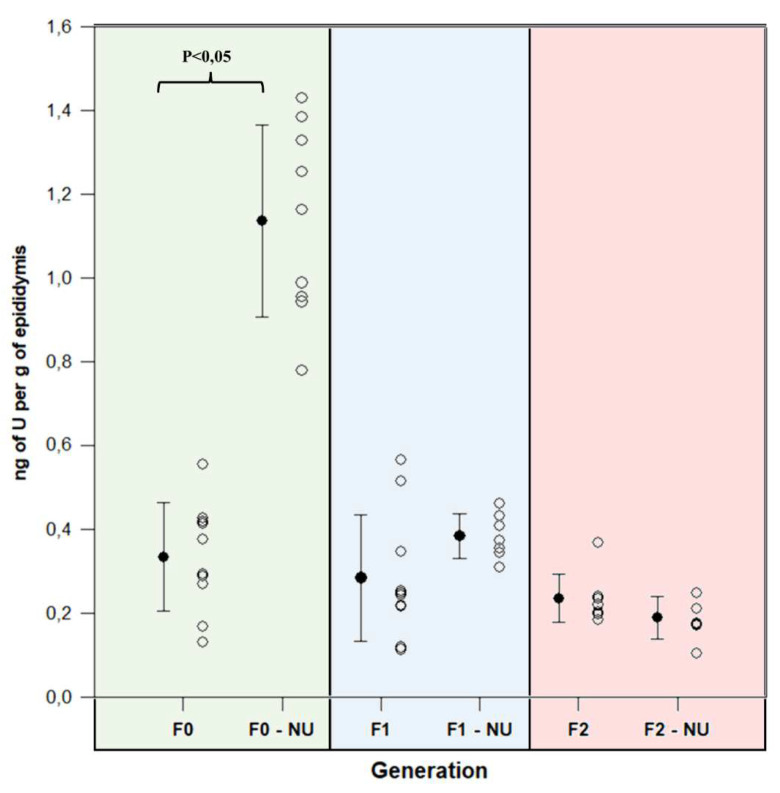
Quantification of uranium in the epididymis for each generation. Significant effects are defined as *p* < 0.05; n = 7–10 per group.

Epididymides were significantly contaminated in generation F0 in the group exposed to uranium compared with the CTRL group (*p* < 0.05) (Figure 2). In this reproductive organ in the former group, the uranium content was 1.14 ± 0.23 g of uranium per gram of tissue. No significant uranium content was detected in generations F1 and F2.

### 2.3. Differences in the NU-Exposed Group in Each Generation

**Figure 3:**
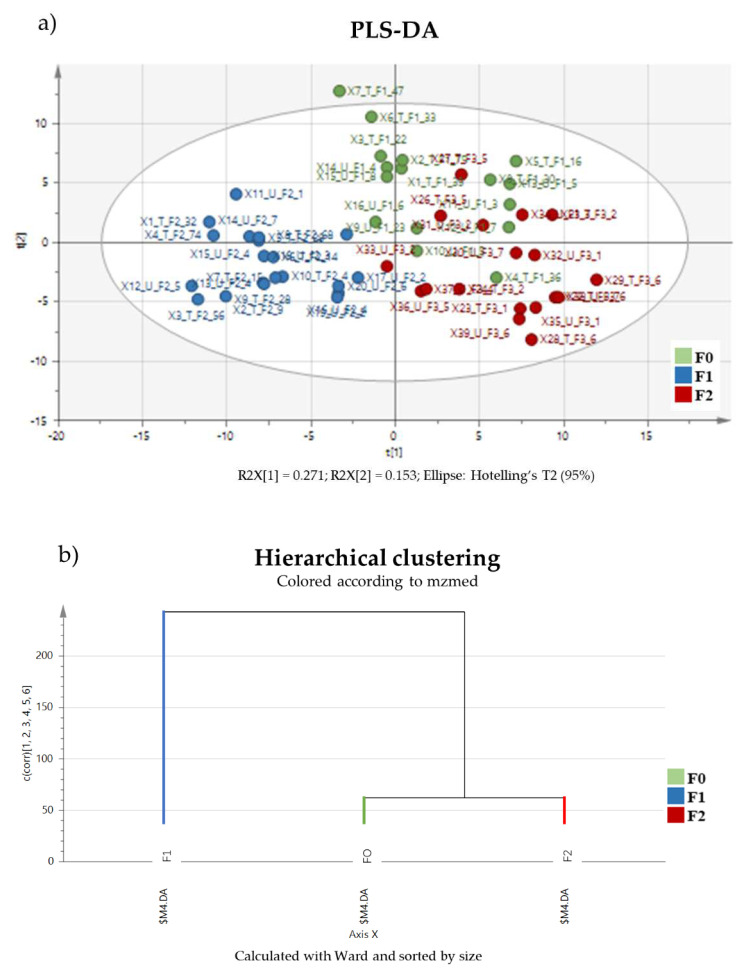
(**a**) PLS-DA model calculated using spermatozoa samples (control and NU-exposed) collected from all generations. The PLS-DA showed significant separation between the metabolomic profiles of each generation (CV-ANOVA; *p* = 2.19 × 10^−27^; R2Y = 96.3; Q2Y = 88.5) (**b**) Hierarchical clustering shows more similarity between generations F0 and F2 than between F0 and F1.

Data observed from spermatozoa extracts were analyzed using different PLS-DA models. The first model was calculated using the matrix containing control and NU-exposed samples from three generations using generation as a dummy matrix. It showed a significant difference between generations (CV-ANOVA; *p* = 2.19 × 10^−27^; R2Y = 96.3; Q2Y = 88.5) (Figure 3).

**Figure 4:**
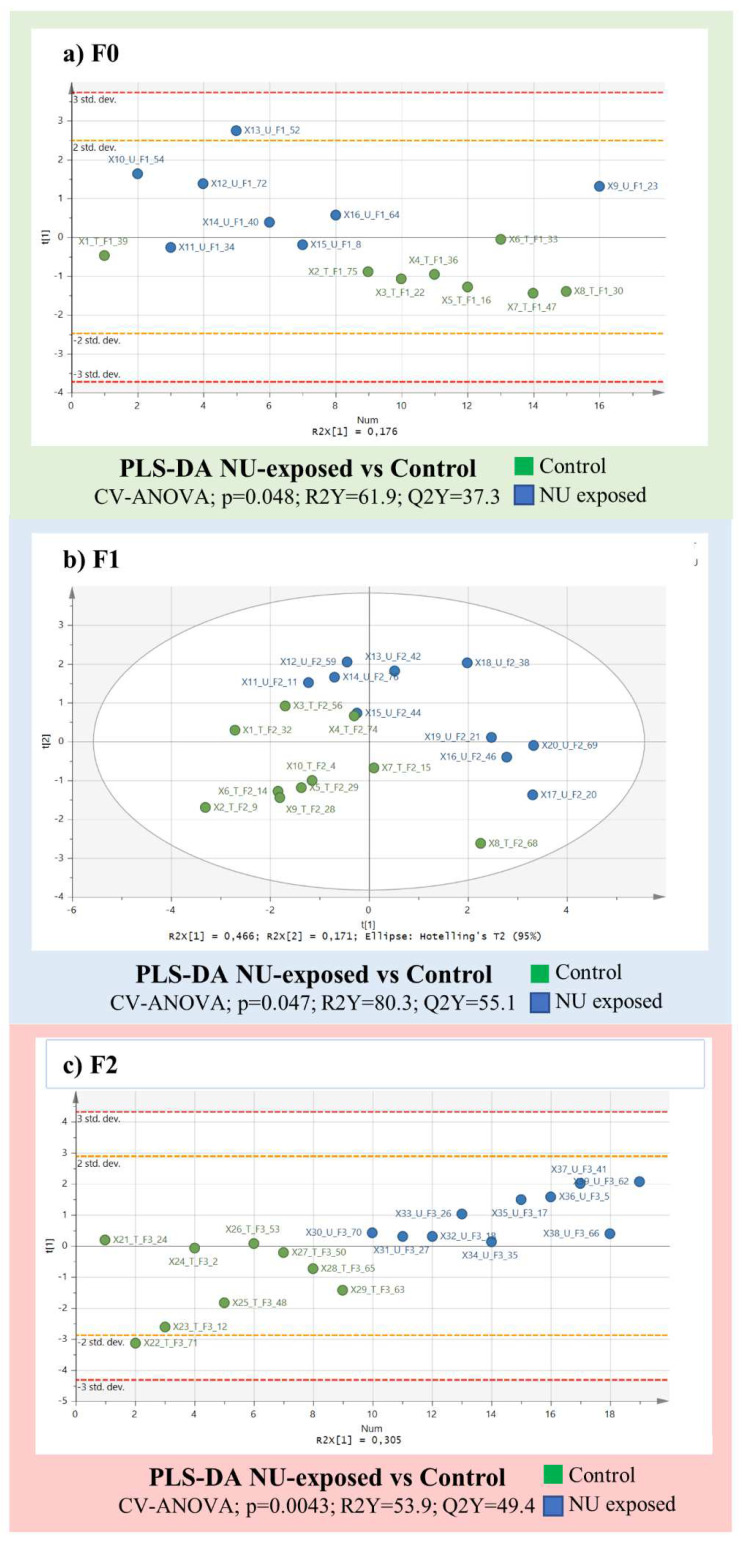
PLS-DA model calculated using spermatozoa samples collected from each generation (control in green and NU-exposed in blue). For generations F0 (**a**) and F2 (**c**), only one component was used, however, for generation F1 (**b**), two components were used for analysis.

Furthermore, NU exposure was evaluated independently in three generations. PLS-DA models were created with no or with different ways of selecting variables to find a model which was able to differentiate between exposed and non-exposed individuals (Figure 4). A PLS-DA for generation F0 containing 48 putatively-identified metabolites did not clearly differentiate between exposed and non-exposed individuals.

Only a selection of 10 metabolites based on VIP > 1.1 clearly separated the two groups of individuals (CV-ANOVA; *p* = 0.048; R2Y(cum) = 61.9%; Q2(cum) = 37.3%) (Figure 4a). Slightly clearer separation was observed in generation F1, where 13 metabolites selected based on their VIP > 1.2) created a PLS-DA model that could be validated (CV-ANOVA; *p* = 0.0047, R2Y(cum) = 80.3%; Q2(cum) = 55.1%) (Figure 4b). Regarding generation F2, a PLS-DA model could only be validated after selecting variables according to coefficients, after selecting seven metabolites as significant (Figure 4c). A validated model (CV-ANOVA; *p* = 0.0043; R2Y(cum) = 53.9%; Q2(cum) = 49.4%) clearly separated NU-exposed from non-exposed individuals.

### 2.4. Identification of Metabolites Common between Generations F0, F1 and F2

**Figure 5:**
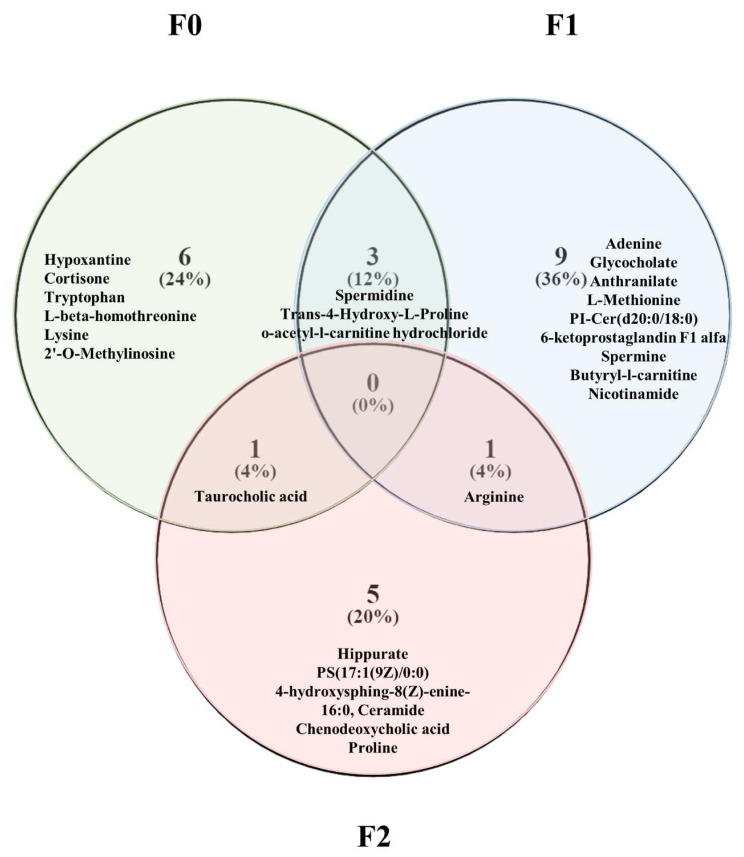
Number (percentage) and name of the most discriminant metabolites identified in spermatozoa samples from each generation used in PLS-DA models.

None of the selected variables was common to the three generations, indicating high multi-generational dimorphism related to NU exposure (Figure 5). However, three metabolites were common to generations F0 and F1: spermidine, Trans-4-hydroxy-L-Proline and 0-acetyl-carnitine hydrochloride. Generations F0 and F2, and generations F1 and F2, shared one common metabolite each, i.e., taurocholic acid and arginine, respectively.

### 2.5. Main Metabolites and Related Pathways in Each Generation

The main metabolic pathways impacted by uranium exposure were analyzed based on the most discriminant metabolites in each generational group. Such analyses showed that some of these pathways could be affected over several generations (Table 1).

## 3. Discussion

Infertility is a major health issue worldwide, and the impact of environmental exposure to uranium on male fertility needs to be studied in more detail. Various biological effects have already been demonstrated for the male and female reproductive functions and fertility [8,9]. The impact of uranium on human infertility is a major concern. Skuhn et al. showed more significant measurable uranium concentrations in the seminal fluid than in the blood of Lebanese male partners in heterosexual couples [40]. They highlighted that significant associations of seminal uranium levels were observed with progressive sperm motility and viability below control levels, and normal morphology. More recent findings revealed semen uranium concentrations in Gulf War Veterans exposed to depleted uranium, and urinary uranium concentrations in women associated with exposure during pregnancy and decreased gestational age and increased risk of preterm birth [41,42]. Our recent experimental research into male fertility highlighted that lifelong exposure to uranium, as a chemical endocrine disruptor, induces subtle testicular and hormonal defects [11]. After multigenerational exposure, we showed that uranium can induce morphological sperm defects and changes in the DNA methylation level [13]. In this paper, we also showed that after the same multigenerational exposure to uranium, significant uranium content was observed in generation F0, with a reduction in the pregnancy rate only detected in generation F1, but without any effects on number of pups per litter and male/female ratio. Together, these results suggest that uranium affects reproductive functions in subsequent generations.

Regarding the risk of heritable effects of such exposure, recent studies have shown differences in global DNA methylation levels in the gonads of exposed individuals at different developmental stages, but also in other organs such as the kidneys [12,43]. These result show, on the one hand, that chronic exposure to low doses of NU can modify the DNA methylation profile of kidney cells (main toxicological target of uranium), but also that uranium could have a genetic effect on the gonads of adult males, even if they are only exposed during the embryonic and fetal periods of generation F0 and the germinal period for the animals in generation F1. Together, these epigenetic effects attest to an early effect occurring during the prenatal development periods and the de novo programming phase of germline and somatic cells. They could also affect the metabolism and physiology of certain organs, including the reproductive organs, and the metabolism of spermatozoa later in life [44,45,46].

It would therefore be worthwhile to expand these results by analyzing the spermogram of the animals in order to identify possible fertility defects, and to analyze the metabolic imprints which may reflect the phenotype of a living organism at a given moment in time. Thanks to its extreme sensitivity and large observational scale, metabolomic analysis can be used to identify subtle-to-significant metabolic modifications that can lead to acute later physiological disorders and health effects [47]. In this field, metabolomics have already been used to study the effects of low-dose incorporated uranium at the systemic and renal levels by analyzing urinary and plasma profiles and renal tissue from contaminated rats [37,48]. More generally, as suggesting by Engel, 2019, using epididymal spermatozoa for metabolomics studies is highly recommended for the purposes of identifying potential biomarkers and developing diagnosis tests for detecting the main failings of the experimental model and potentially infertile men [49]. In fact, the metabolomic analysis of epididymal spermatozoa could be used to highlight discriminating profiles in contaminated individuals [26].

Based on the PCA analysis of the annotated matrices from the sperm samples from the three generations in general, initial observations yielded different profiles for each generation without any detectable effect that was attributable to uranium. It is interesting to note that sperm metabolism was not constant, and that intergenerational fluctuation was much greater than that found for uranium effects, confirming the low-dose range of this study (as previously shown with aging in urine [48]). At this dose level, the effect of uranium seemed to be weak (Figure 4). If we consider each generation individually, the PLS discriminant analysis of the 48 annotated variables from the data row matrix could be used to obtain predictive models for the 10 metabolites identified from generation F0, 13 in generation F1 and 7 in generation F2 (Table 1). Despite the weak effect of the uranium exposure, when focusing on the variance specifically linked to treatments based on the selected variables, and the most sensitive variables to uranium exposure in each generation (PLS-DA analyses, Table 1), the effect of uranium was not negligible, i.e., it represents 37%, 58%, and 49% of this “sub” metabolome variation in generations F0, F1 and F2, respectively. 

**Figure 6:**
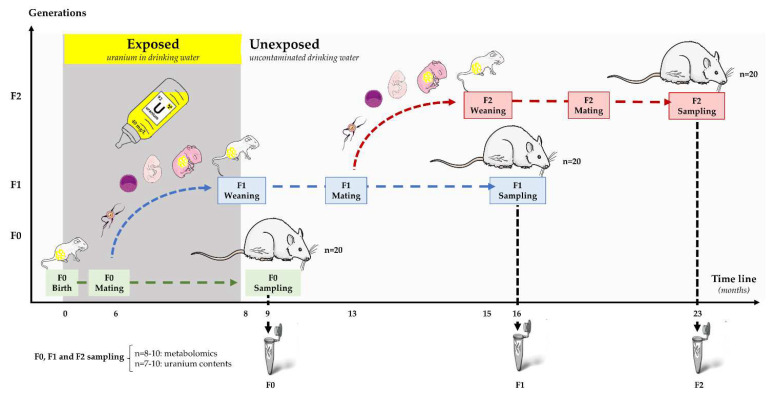
Multigenerational uranium exposure protocol. Three generations of male and female rats (F0, F1 and F2) (n = 20) were monitored. Generation F0 was exposed over 9 months from birth through drinking water with natural uranium (NU). Control animals drank uncontaminated mineral water. Generation F1 was contaminated in utero and through their mothers’ milk (F0) until weaning. After generation F1 was weaned, contamination was stopped, and all groups of rats were provided with uncontaminated mineral drinking water ad libitum. Generation F2 was only exposed to uranium from parental (F1) germ cells.

The results therefore confirm that parental exposure during both the preconceptional and prenatal periods can have metabolic effects on the spermatozoa for the next two generations. The observation of the 3 discriminant metabolomic profiles reveals that each generational effect differed from the others with only 12% overlap between the first and second generations and 4% between both the second and third generations, and between the third and first generations. No characteristics seemed to be common to all three generations (Figure 6).

In order to search for predictive indicators of reproductive function impairment, the observation of the main impacted metabolites and associated metabolisms showed effects on purine and steroids metabolisms. Among these, the metabolism of purines are involved in cellular translation processes and sperm motility for generation F1 [50,51]. Glucocorticoids involved in steroidogenesis [52], but also in sugar metabolism, immune function and inflammatory processes, appears as a discriminant metabolite in this first generation [53].

Other metabolism such as energy metabolism could be impacted in generations F0, F1 and F2, in which level of carnitines, implied in the transport of fatty acids to the mitochondria, are found to be affected by uranium [54,55]. They also play an antioxidant role in the lipoperoxidation of membrane phospholipids, which helps to regulate oxidative stress and influence male fertility (apoptosis, sperm parameters and function) [56,57]. 

Uranium also seems to have an effect on polyamine metabolism, involving different metabolites such as spermidine (F0) and L-methionine (F1), spermine and finally arginine (F1, F2) [58]. Polyamines are involved in many processes which are essential for cell growth, DNA double helix stabilization and cationic transporters [59,60,61,62,63]. For example, arginine is known to be involved in the anti-inflammatory process, but also, in the sperm motility [64].

Bile acids are also part of the discriminant metabolites found in sperm for generations F0, F1 and F2. They are also involved in energy metabolism, steroidogenesis and in testicular defects reducing fertility [65,66]. For each of the three generations, proline also plays an important role in fertility because it protects spermatozoa from free radical damage by stabilizing the membrane structure, reduces lipid peroxidation and improves sperm motility [67,68].

In addition, tryptophan and the nicotinate-nicotinamide pathways, associated with NAD+ production, are already known to be impacted by exposure to uranium in the kidneys [37,69]. They are involved in the redox system, and in the inflammatory processes of the organism. The deregulation of these pathway in sperm shows that it is not specific to kidney. Finally, other discriminant metabolites as ceramides, and specifically lysophosphatidyl serine are involved in inflammatory processes and in sperm motility [70,71].

In conclusion, based on our metabolomic results, we demonstrated that metabolomics applied to epididymal spermatozoa offers a novel approach to detecting the multigenerational metabolomic profiles induced by uranium in an experimental model. For the first time, dysregulation of tryptophan and nicotinamide pathways is also detected in sperm, and new markers specific to sperm metabolome exposed to uranium are highlighted, as spermine and cholic acid. In our multigenerational uranium exposure model and regarding the pregnancy rate observed in generation F1, these metabolomic results suggested some specific pathway affected in sperm for F1 generation. More particularly, in this in utero exposed generation, which is especially sensitive, we detected discriminant metabolites involved in sperm motility, energy metabolism, steroidogenesis, cell growth and DNA molecule stabilization. To complete these new findings and our recent results showing sperm morphological defect [13], future testicular studies on steroidogenesis, spermatogenesis and sperm motility could improve our understanding of how uranium could affect fertility.

## 4. Materials and Methods

### 4.1. Experimental Procedure and Sample Collection

Outbred Sprague–Dawley, 12-week-old and 16-day pregnant, female rats (parents’ generation) were obtained from Charles River Laboratories (L’Arbresle, France). They were housed individually and maintained in a 12 h light/12 h dark cycle (regular cycle) at 21 °C and 50% humidity, with ad libitum access to a standard rodent pellet diet and water until birth. All experimental procedures were approved by the Animal Care Committee of the Institute of Radioprotection and Nuclear Safety (IRSN, Fontenay-aux-Roses, France) and complied with French regulations on animal experimentation (French Ministry of Agriculture Act No. 87-848, 19 October 1987, modified 20 May 2001).

The multigenerational study design is already described in [12,43] (Figure 6). In summary, the present protocol includes three generations (F0, F1 and F2) of male and female rats (n = 20). Exposure to Natural Uranium (NU) started from birth and continued up to the age of 9 months in generation F0 (offspring of the parents’ generation). Generation F0 was mostly exposed to NU through lactation (human offspring absorbs approximately 5% of the mother’s daily uranium dose) and contaminated drinking water. All control groups of rats received uncontaminated drinking water *ad libitum*. F1 rats were contaminated in utero and through lactation until weaning [72]. After weaning, they drank uncontaminated mineral water. The last generation, F2, only received mineral water. Generation F2 was only exposed to uranium from parental (F1) germ cells.

NU (Mc Arthur) was obtained from CERCA (Pierrelatte, France) in the form of uranyl nitrate hexahydrate (UO_2_ (NO_3_)_2_ 6H_2_O) was prepared to obtain a final uranium concentration in the drinking solution was 40 mg L^−1^ of mineral water obtained from Evian® (Evian-les-Bains, France), which resulted in a daily uranium intake dose of 1 mg/rat/day) [73]. The specific activity of the NU was 2.42 10^+4^Bq g^−1^, and the isotopic compositions were ^238^U~99.307%, ^235^U~0.688%, and ^234^U~0.005%. This NU concentration was three times higher than the highest uranium concentration, i.e., 12.4 mg L^−1^ naturally found in Finnish wells [74], half of the WHO 2011 drinking-water guideline for uranium, defined as 0.030 mg L^−1^ [75], and not nephrotoxic [76]. 

In each generation, 9-month-old rats were deeply anesthetized by inhaling 5% isoflurane (Abbot France, Rungis, France) and euthanized by an intracardiac puncture. 

The epididymis (n = 8–10 per group/per generation) was deep-frozen in liquid nitrogen and stored at −80 °C for metabolomic analysis. Other epididymis samples (n = 7–10 per group) were weighed and stored at –20 °C to determine uranium content.

### 4.2. Uranium Content

Epididymis was prepared by adding 8 mL of ultrapure 69% nitric acid (ARISTAR, VWR, France) and 2 mL of hydrogen peroxide 30% and mineralizing them in a 1000 W microwave (Ethos Touch, Milestone Microwave Laboratory Systems, Italy) with a 20-min. temperature increase to 180 °C and then a steady phase of 10 min. at 180 °C. The uranium content of mineralized samples was determined with an inductively coupled plasma mass spectrometer (ICP-MS Xseries 2, ThermoElectron, France) using bismuth (1 mg L^−1^) as the internal standard. The ICP-MS detection limit for uranium is 1 ng∙L^−1^. Values were expressed as ng U per g of tissue.

### 4.3. Metabolomic Study

#### 4.3.1. Sample Preparation

After thawing at room temperature, the whole epididymis was homogenized in medium M199 (Sigma-Aldrich, St. Louise, MO, USA). Ten cycles of a manual Potter–Elvehjem homogenizer were used for each sample to isolate epididymal spermatozoa. The cells were counted on the Malassez counting chamber to determine the sperm concentration and the purity of the sample. The concentration of epididymal cells was less than 8%. Each sample was normalized to obtain a concentration of 1.3 × 10^−7^ sperm in 250 µL of medium M199.

Experiments were performed on eight replicates (F0) and ten replicates (F1 and F2). Sperm samples were vortex mixed to homogenize. Then, 500 µL of 80% cold (−80 °C) methanol was added to 100 µL of sample and agitated slowly before a one-hour incubation period at −80 °C. After one-minute of vortex mixing, samples were centrifuged for 15 min at 4 °C and 11,000 RPM and 450 µL of supernatant was evaporated under the gentle nitrogen steam and suspended in 100 µL water/acetonitrile/formic acid, 90/10/0.1, (*v*/*v*/*v*). Then, 20 µL of each sample was collected to obtain a pooled sample for use as a quality control. The mixture used to dissolve dry extracts was also used as blank sample.

#### 4.3.2. Ultra-High-Performance Liquid Chromatography-High Resolution Mass Spectrometry

High performance liquid chromatography coupled with high-resolution mass spectrometry was used for sample analysis. Chromatographic separation was carried out using a Dionex UltiMate 3000 (Thermo Fisher Scientific). First, 5 µL of each sample was injected into a reverse phase Hypersil Gold C18 (100 mm × 2.1 mm × 1.9 µm) (Thermo Scientific, France) column kept at 40 °C. The flow rate was maintained at 400 µL/mL, and 0.1% formic acid solutions in water and acetonitrile were used in mobile phases A and B respectively. A first minute at 0% of B in the isocratic elution was followed by ten minutes on a linear gradient to 100% B, which was then maintained in isocratic mode for two minutes. Initial conditions were recreated in one minute following two minutes of column equilibration.

High resolution mass spectrometry analysis was performed using the Q-Exactive Plus hybrid mass spectrometer (Thermo Fisher Scientific, Bremen, Germany) with a Heated Electrospray Ionization (H-ESI II) probe working in positive and negative ionization modes. Ionization conditions were as follows: spray voltage, ±3500 V, transfer capillary temperature, 320 °C, sheath and auxiliary gas flow rates, 30 and 8 arbitrary units respectively and gas temperature 310 °C. Ion transfer was maintained keeping S-lens RF at 55 V. Mass spectra were acquired in the 80–1000 *m*/*z* range, with maximal injection time 250 ms and resolving power set to 35,000 FWHM (Full Width Half Maximum) for the theoretical *m*/*z* 200. Instrument setup was controlled using Thermo Xcalibur 3.0.63 software and the mass spectrometer was controlled using the Tune Q Exactive Plus 2.5 application.

Samples were analyzed in one analytical batch. First, the blank samples were analyzed with five replicates, followed by ten pool samples used to equilibrize the analytical system. Samples were then analyzed in a random order interspaced by one pool sample every five samples. At the end of the analytical batch, a pool sample was tested using High Collision Dissociation (HCD) and a Data-Dependent Analysis to obtain the MS/MS spectra and elucidate the structure of the many metabolites.

#### 4.3.3. Data Pre-Processing

The ProteoWizard application was used to convert raw spectra into separated positive/negative mzXML files. Only positive ionization spectra were processed. The XCMS library under the R environment was used to extract data using the following parameters: peak detection method—centWave; peak width—2 to 15 s; S/N threshold—3; noise —10000; *m*/*z* tolerance between two consecutive scans—5 ppm. The prefilter for the peaks detected was set to four consecutive scans with intensities higher than 100,000. The peaks of samples were aligned and grouped using Obiwarp and density methods, respectively.

Extracted data matrices were then filtered to eliminate the analytical background and correct for analytical drift. Analytical batch drift was corrected using the Van der Kloet algorithm. Blank samples were used to filter data from instrumental noise, and pool samples to filter signals that varied by more than 30%. The final matrix contained 555 ions and was used for the untargeted metabolomic analysis.

### 4.4. Statistical Analysis

#### 4.4.1. Uranium Content, Fertility Parameters

Uranium concentration results are expressed as mean ± standard deviation (SD). Kruskal-Wallis One-way ANOVA was performed, and Dunn’s Method was used for all pairwise multiple comparisons. The Fisher exact test was used to compare pregnancy rate, number of pups per litter and the male/female ratio. Differences were considered statistically significant when *p* < 0.05 (Sigmaplot Stat software, SPSS, Paris, France).

#### 4.4.2. LC-MS Data Analysis

Multivariate statistical analyses were performed using SIMCA-P 14.0 software (Umetrics, Sartorius, France) and partial least squares discriminant analysis (PLS-DA) models were obtained after transforming log 10 [1 + 10^4^] data and Pareto normalization. Model were validated by CV-ANOVA and permutation tests.

#### 4.4.3. Variable Selection and Metabolite Identification

Discriminant metabolites were selected according to their variable importance in projection (VIP) score SIMCA P algorithm. Normal probability plot (NNP) distribution was used to determine the appropriate threshold for significance. Variables were putatively-identified using the laboratory data base, Metlin and MZedDB database browsers (Aberystwyth University, Aberystwyth, UK) freely available online [77], according to chemical formulas generated from mass measurements (error < 5 ppm). Full MS and MS/MS spectra were compared for standard chemical samples, biological samples, and spectral databases (mainly laboratory data base, HMDB, Metlin, and MassBank) to identify metabolites.

## Figures and Tables

**Table 1:** Main related pathways identified from the most discriminant metabolites in each generation.

	Generations		
Metabolisms	F0	F1	F2
**Purines**		Adenine	
	2′-O-Methylinosine		
	Hypoxanthine		
**Glycocorticoïdes**	Cortisone		
**Carnitines**	Lysine		
	**o-acetyl-l-carnitine hydrochloride**	**o-acetyl-l-carnitine hydrochloride**	
		Butyryl-l-carnitine	
**Polyamines**		L-Methionine	
	**Spermidine**	**Spermidine**	
		Spermine	
		**Arginine**	**Arginine**
**Bile acids**	**Taurocholic acid**		**Taurocholic acid**
		Glycocholic acid	
			Chenodeoxycholic acid
**Proline**	**Trans-4-hydroxy-L-proline**	**Trans-4-hydroxy-L-proline**	
			Proline
**Tryptophan**	Tryptophan		
		Anthranilate	
**Nicotinate-nicotinamide**		Nicotinamide	
**Ceramides**		PI-Cer(d20:0/18:0)	
			4-hydroxysphing-8(Z)-enine-16:0, ceramide
**Phospholipids**			PS(17:1(9Z)/0:0)
**Prostacyclin**		6-ketoprostaglandin F1 alfa	
**Microbiotic origin, glucid metabolism**			Hippurate
**Unknow origin**	L-beta-homothreonine		

## Data Availability

Data are available from the corresponding authors upon request.
